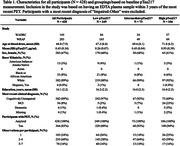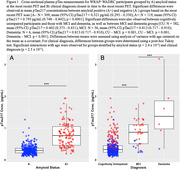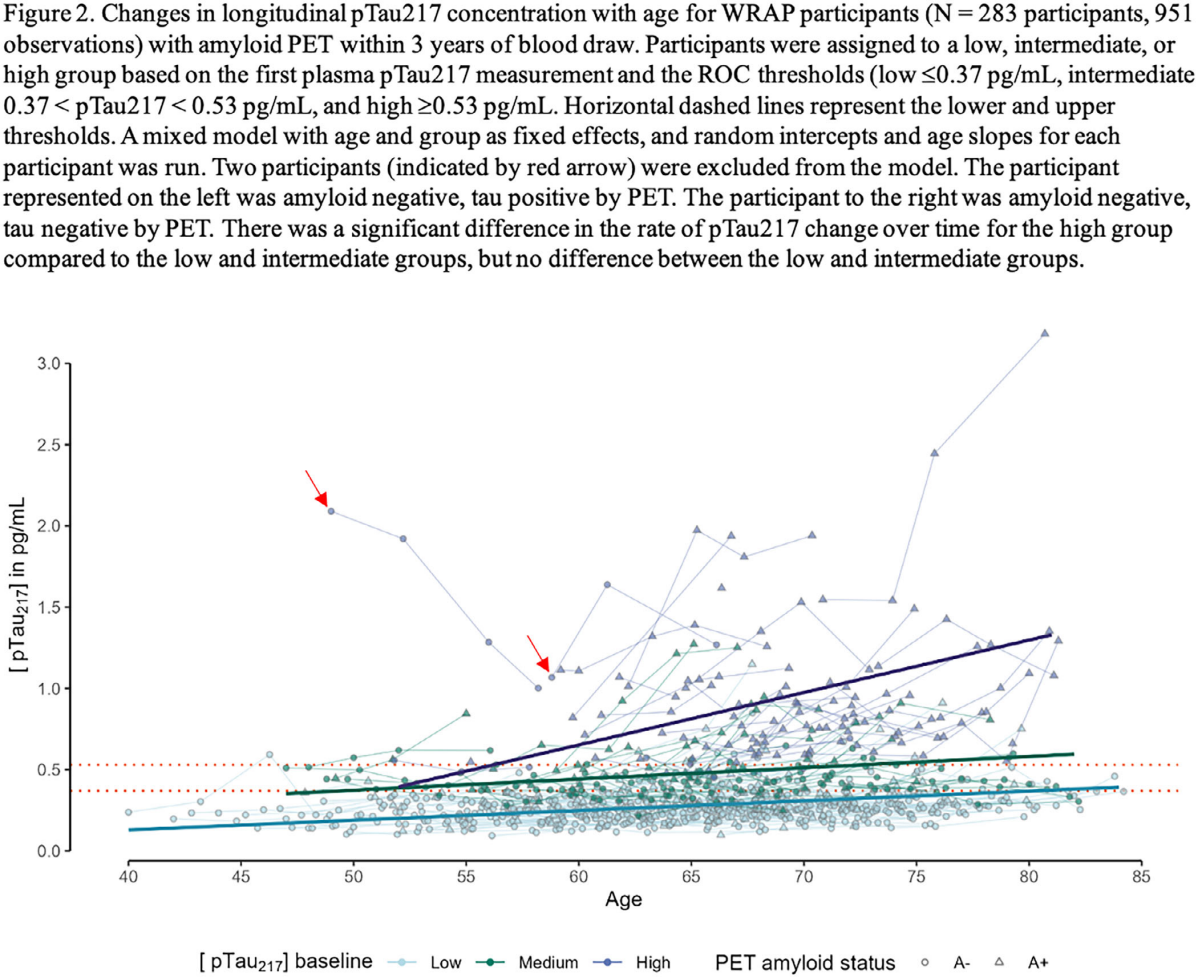# Longitudinal Plasma pTau217 Measurements in Preclinical Alzheimer’s Disease

**DOI:** 10.1002/alz.091896

**Published:** 2025-01-09

**Authors:** Rachael E Wilson, Ramiro Eduardo Rea Reyes, Rebecca E. Langhough, Erin M. Jonaitis, Lianlian Du, Emily Legois, Elysse Keske, Aaron Fredricks, Martie Marshall, Cindy Jensen, Monica VandenLangenberg, Beckie Jeffers, Bailey Wheelock, Max Mershon, Jo Kaseno, Tobey J. Betthauser, Bradley T. Christian, Ozioma C Okonkwo, Barbara B. Bendlin, Corinne D. Engelman, Carey E. Gleason, Kirk J. Hogan, Nathaniel A. Chin, Cynthia M. Carlsson, Sanjay Asthana, Sterling C. Johnson, Henrik Zetterberg

**Affiliations:** ^1^ University of Wisconsin‐Madison, Madison, WI USA; ^2^ Wisconsin Alzheimer’s Institute, University of Wisconsin School of Medicine and Public Health, Madison, WI USA; ^3^ Wisconsin Alzheimer’s Institute, University of Wisconsin‐Madison School of Medicine and Public Health, Madison, WI USA; ^4^ University of Wisconsin‐Madison, School of Medicine and Public Health, Madison, WI USA; ^5^ University of Wisconsin, Madison, WI USA; ^6^ Wisconsin Alzheimer's Institute, University of Wisconsin, Madison, WI USA; ^7^ School of Medicine and Public Health, University of Wisconsin‐Madison, Madison, WI USA; ^8^ Alzheimer’s Disease Research Center, University of Wisconsin School of Medicine and Public Health, Madison, WI USA; ^9^ Anesthesiology, University of Wisconsin School of Medicine and Public Health, Madison, WI USA; ^10^ University of Wisconsin‐Madison School of Medicine and Public Health, Madison, WI USA; ^11^ Wisconsin Alzheimer's Disease Research Center, University of Wisconsin School of Medicine and Public Health, Madison, WI USA; ^12^ Wisconsin Alzheimer's Institute, Madison, WI USA; ^13^ Wisconsin Alzheimer's Disease Research Center, School of Medicine and Public Health, University of Wisconsin‐Madison, Madison, WI USA; ^14^ Wisconsin Alzheimer's Disease Research Center, Madison, WI USA; ^15^ Hong Kong Center for Neurodegenerative Diseases, Hong Kong China; ^16^ UCL Institute of Neurology, Queen Square, London UK; ^17^ Department of Psychiatry and Neurochemistry, Institute of Neuroscience and Physiology, The Sahlgrenska Academy at the University of Gothenburg, Mölndal Sweden

## Abstract

**Background:**

Plasma pTau217 (tau phosphorylated at threonine 217) assays will expand access to screening for Alzheimer’s disease (AD). However, clinical interpretation is not well‐established, particularly during the preclinical window when interventions may be most effective. Using plasma samples from primarily late‐midlife, cognitively unimpaired Wisconsin Registry for Alzheimer’s Prevention (WRAP) and Wisconsin Alzheimer’s Disease Center (WADRC) participants, we investigated pTau217 agreement with amyloid and tau PET then compared trajectories between participants grouped by baseline pTau217.

**Methods:**

EDTA plasma samples from 428 participants were analyzed using the ALZpath pTau217 Simoa assay on a Quanterix HD‐X (Table 1). Amyloid and tau positivity were defined as global [^11^C]‐PiB DVR>1.19 (21.6 centiloids) and [^18^F]‐MK6240 temporal meta‐ROI SUVR>1.3, respectively. In separate receiver operating characteristic (ROC) analyses using pTau217 to classify amyloid and tau PET, the area under the curve (95% CI) and optimal (Youden) cutoffs were, respectively: 0.91 (0.87‐0.94), 0.37 pg/mL; and 0.89 (0.83‐0.94), 0.53 pg/mL. To characterize pTau217 rates of change based on baseline measurements, participants were clustered into low (≤0.37 pg/mL), intermediate (0.37 < pTau217 < 0.53 pg/mL), and high (≥0.53 pg/mL) groups according to the ROC thresholds. Linear mixed models were then run with group and age as fixed effects and including random person‐level intercept and age slopes.

**Results:**

Mean pTau217 concentration was 2.5 times higher for amyloid positive participants compared to amyloid negative (Figure 1A; p < 0.001). Significant differences were also observed between CU, MCI, and dementia groups (Figure 1B; CU‐MCI: p < 0.001, CU‐MCI: p<0.001, Dementia‐MCI: p<0.001). Mixed effects models indicated that pTau217 increased at a higher rate in the high group compared to other groups (Figure 2; simple slopes low‐high: t(361)=‐7.010, p<0.001, intermediate‐high: t(318)=‐5.712, p < 0.001). No differences were observed between simple slopes for low and intermediate groups (t(235)=‐0.314, p=0.9470).

**Conclusion:**

These results suggest that plasma ALZpath pTau217 is a very good proxy for molecular PET for detecting AD pathology prior to symptoms. Strategies such as assigning an indeterminate zone for secondary confirmation (Brum, W., et al, 2023) would improve accuracy and lessen the burden on existing resources while expanding access to the broader community.